# Impact of somatic mutations on prognosis in resected non‐small‐cell lung cancer: The Japan Molecular Epidemiology for lung cancer study

**DOI:** 10.1002/cam4.2897

**Published:** 2020-02-05

**Authors:** Akihiro Tamiya, Yasuhiro Koh, Shun‐ichi Isa, Akihito Kubo, Masahiko Ando, Hideo Saka, Naoki Yoshimoto, Sadanori Takeo, Hirofumi Adachi, Tsutomu Tagawa, Osamu Kawashima, Motohiro Yamashita, Kazuhiko Kataoka, Mitsuhiro Takenoyama, Yukiyasu Takeuchi, Katsuya Watanabe, Akihide Matsumura, Tomoya Kawaguchi

**Affiliations:** ^1^ National Hospital Organization (NHO) Kinki‐Chuo Chest Medical Center Sakai Japan; ^2^ Wakayama Medical University Wakayama Japan; ^3^ Aichi Medical University School of Medicine Nagakute Japan; ^4^ Nagoya University Hospital Nagoya Nagoya Japan; ^5^ NHO Nagoya Medical Center Nagoya Japan; ^6^ Isikiriseiki Hospital Higashiosaka Japan; ^7^ NHO Kyushu Medical Center Fukuoka Japan; ^8^ NHO Hokkaido Cancer Center Sapporo Japan; ^9^ NHO Nagasaki Medical Center Omura Japan; ^10^ NHO Shibukawa Medical Center Shibukawa Japan; ^11^ NHO Shikoku Cancer Center Matuyama Japan; ^12^ NHO Iwakuni Clinical Center Iwakuni Japan; ^13^ NHO Kyushu Cancer Center Fukuoka Japan; ^14^ NHO Toneyama Medical Center Toyonaka Japan; ^15^ NHO Yokohama Medical Center Yokohama Japan; ^16^ Graduate School of Medicine Osaka City University Osaka Japan

**Keywords:** next‐generation sequencing, non‐small cell lung cancer, overall survival, recurrence free survival, somatic mutation

## Abstract

**Background:**

To report the follow up data and clinical outcomes of the JME study (UMIN 000008177), a prospective, multicenter, molecular epidemiology examination of 876 surgically resected non‐small‐cell lung cancer (NSCLC) cases, and the impact of somatic mutations (72 cancer‐associated genes) on recurrence‐free survival (RFS) and overall survival (OS).

**Methods:**

Patients were enrolled between July 2012 and December 2013, with follow up to 30th November 2017. A Cox proportional hazards model was used to assess the impact of gene mutations on RFS and OS, considering sex, smoking history, age, stage, histology, *EGFR*, *KRAS*, *TP53*, and number of coexisting mutations.

**Results:**

Of 876 patients, 172 had ≥2 somatic mutations. Median follow‐up was 48.4 months. On multivariate analysis, number of coexisting mutations (≥2 vs 0 or 1, HR = 2.012, 95% CI: 1.488‐2.695), age (≥70 vs <70 years, HR = 1.583, 95% CI: 1.229‐2.049), gender (male vs female, HR = 1.503, 95% CI: 1.045‐2.170) and pathological stage (II vs I, HR = 3.386, 95% CI: 2.447‐4.646; ≥III vs I, HR = 6.307, 95% CI: 4.680‐8.476) were significantly associated with RFS, while *EGFR* mutation (yes vs no, HR = 0.482, 95% CI: 0.309‐0.736), number of coexisting mutations (≥2 vs 0 or 1, HR = 1.695, 95% CI: 1.143‐2.467), age (≥70 vs <70 years, HR = 1.932, 95% CI: 1.385‐2.726), and pathological stage (II vs I, HR = 2.209, 95% CI: 1.431‐3.347; ≥III vs I, HR = 5.286, 95% CI: 3.682‐7.566) were also significant for OS.

**Conclusion:**

A smaller number of coexisting mutations, earlier stage, and younger age were associated with longer RFS and OS, while *EGFR* mutations were significantly associated with improved OS.

## Graphical abstract

A prospective, multi‐center, molecular epidemiology study of 876 surgically resected non‐small cell lung cancer cases, and the impact of somatic mutations (72 cancer‐associated genes) on recurrence‐free survival and overall survival (OS). A smaller number of co‐existing mutations, earlier stage, and younger age were associated with longer recurrence free survival and OS, while epidermal growth factor receptor mutations were significantly associated with improved OS.

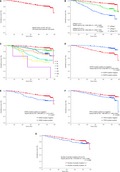

## 1 INTRODUCTION

Lung cancer is the leading cause of cancer‐related morbidity and death worldwide and is one of the most molecularly complex cancers.^1^, ^2^, ^3^ Driver mutations in cancers have been intensively examined and identified over a decade using advanced and robust tools, namely, next‐generation sequencing (NGS), and these serve as the basis for the precision therapy.^4^ Certainly, some of these somatic mutations play a critical role in cancer development, and a molecular epidemiological approach has been helpful to uncover the mechanisms of the disease and provide a strategy for cancer prevention. However, a number of genetic changes may not have functional importance, and it seems a few meaningful driver mutations have a prognostic or predictive value.

Among the genes responsible for somatic mutations in non‐small‐cell lung cancer (NSCLC), the most frequent driver oncogenes were epidermal growth factor receptor (*EGFR*), v‐Ki‐ras2 Kirsten rat sarcoma (*KRAS*), and tumor protein p53 (*TP53*). Sensitizing *EGFR* mutations were first reported in 2004^5^ and have become the most important somatic mutations for precision therapy for advanced NSCLC because of their high prevalence and the striking treatment efficacy of *EGFR* tyrosine kinase inhibitors (TKIs).^6^, ^7^, ^8^ *KRAS*, a member of the RAS family, was one of the first oncogene to have been identified in NSCLC.^9^, ^10^ *KRAS* mutations occur frequently in codons 12 and 13,^11^ are usually found in nonsquamous carcinoma and in patients who smoke, and are associated with a poor prognosis.^12^, ^13^ *TP53* (in relation to mutations in the tumor suppressor gene that encodes p53 protein) has a high detection rate in all subtypes of lung cancer, with reported mutation incidence of approximately 40%‐80%.^14^ Although *TP53* plays multiple roles in prevention and suppression of abnormal cell growth through cell cycle arrest, the prognostic or predictive effect of *TP53* in NSCLC is limited.^15^, ^16^ Furthermore, the frequency of multiple driver mutations, including the three gene mutations mentioned, in NSCLC has not been reported, and the prognostic and predictive effects have not been well studied.

We had previously reported molecular profiling as a primary endpoint in a prospective, multicenter, molecular epidemiology research by collecting samples from 876 patients with NSCLC who had undergone surgical resection and examining the somatic mutations in 72 cancer‐associated genes using next‐generation sequencing (Japan Molecular Epidemiology for lung cancer study [JME]).^17^ In this report, we have demonstrated the incidence of somatic mutation status in resected NSCLC, the mutational spectrum associated with a unique signature of exposure to smoking and body mass index (BMI), and the noteworthy effect of smoking on developing driver mutations.

The secondary endpoints, as per the present research, were overall survival (OS) and recurrence‐free survival (RFS) analyses (UMIN 000008177). Therefore, to clarify the impact of somatic mutations on RFS and OS for resected NSCLC, the follow up data and clinical outcomes of the JME study were collected prospectively, and the impact of somatic mutations, including *EGFR*, *KRAS*, and *TP53* and coexisting multiple mutations, on RFS and OS was analyzed.

## 2 PATIENTS AND METHODS

### 2.1 Patients

Eligible patients had pathologically NSCLC with clinical stage I, II, IIIA or IIIB disease (TNM classification version 7^18^) and had undergone surgery with curative intent. The projected sample size was 900 (450 smokers and 450 nonsmokers) as reported earlier.^17^ Patients with prior radiotherapy and/or chemotherapy were excluded, as were patients with other prior malignancies except for adequately treated basal cell or squamous‐cell skin cancer or in situ cervical cancer. Other criteria for inclusion were the availability of a surgical specimen and written informed consent. All informed consents were obtained before surgery.

### 2.2 Methods of NGS analysis

All formalin fixed paraffin embedded (FFPE) surgical tissues were sent to the central laboratory for genomic analysis and immune‐histochemical staining. DNA was extracted from the FFPE samples, and quality control assessment was performed as reported earlier.^1^ Median tumor cellularity in surgical specimens for molecular estimates was 50% (ranging 10%‐100%). Multiplexed, targeted deep sequencing was used to evaluate tumors. A total of 72 cancer‐associated genes were selected based on previous reports^1^, ^19^ to cover all critical mutations for analysis of prognostic impact, including *TP53*, *ALK*, *EGFR*, *KRAS*, *BRAF*, *RET*, *STK11*, *KEAP1*, *PIK3CA*, *MET*, *RB1*, *ABL1*, *CSF1R*, *FGFR2*, *FGFR3*, *JAK2*, *JAK3*, *NOTCH1*, *AKT1*, *AKT3*, *CTNNB1*, *FLT3*, *NPM1*, *SMAD4*, *GNA11*, *KDR*, *NRAS*, *SMARCB1*, *APC*, *ERBB2*, *ERBB3*, *ERBB4*, *GNAQ*, *KIT*, *PDGFRA*, *SMO*, *ATM*, *GNAS*, *SRC*, *FBXW7*, *HNF1A*, *PTEN*, *CDH1*, *FGFR1*, *FGFR4*, *HRAS*, *MLH1*, *PTPN11*, *CDKN2a*, *IDH1*, *MPL*, *VHL*, *NF1*, *SMARC4*, *ARID1A*, *RBM10*, *SETD1*, *CBL*, *CUL3*, *DDR2*, *RASA1*, *TSC1*, *TSC2*, *CTIF*, *NFE2L2*, *PPP2R1A*, *BRD3*, *CCND1*, *MYC*, *PTCH1*, *U2AF1*, and *MAP2K1*. In addition, *ALK* rearrangements were detected by immunohistochemical staining using (5A4) CD 246 antibody.^20^

### 2.3 Statistical considerations

Clinical data, including sex, smoking history, age, stage, histology, mutations in *EGFR*, *KRAS*, and *TP53* genes, and other minor mutations were used for the this study, as well as additional post hoc analysis on the number of coexisting mutations. Kaplan‐Meier (K–M) plots were used for RFS and OS analyses and for determination of median and 95% CI values. A *P* value of less than .05 was considered significant. Multivariate logistic regression model and Cox proportional hazards models were used to assess the impact of the mutations on RFS and OS. Statistical analysis was conducted using JMP software (version 12, SAS Institute Inc). This study was registered (UMIN 000008177).

## 3 RESULTS

### 3.1 Patients' characteristics

Between July 2012 and December 2013, 957 patients were enrolled from 43 institutions, and, upon performing molecular analyses, and 876 samples were successfully examined for gene mutations by NGS with a mean coverage of 4253×, as reported previously.^17^ All 876 patients' clinical and prognostic data were prospectively collected. The data cut‐off date for this study was November 30th, 2017, and the median follow up time was 48.4 months. The incidence of tumor gene mutations indicated in this study has previously been reported (JME study).^17^ The characteristics of the patients are shown in Table 1. In the present analysis, the median age was 70 years (range 23‐92 years), 450 of 876 patients were 70 years old or older, 457 were female, the majority of patients (734 patients) was diagnosed with nonsquamous cell carcinoma (non‐SQ), and 441 patients had a smoking history. The number of patients according to stage was 618 patients in stage I, 131 in stage II, and 127 in stage III‐IV. Regarding somatic gene alterations, 352 patients had *EGFR* mutations, 235 had *TP53* mutations, 73 had *KRAS* mutations, 34 had ALK rearrangements, and 172 had two or more coexisting mutations.

**Table 1:** Patient characteristics

Characteristic	
Total no.	876
Age (years)	
Median (range)	70 (23‐92)
<70	426
≥70	450
Sex	
Male	419
Female	457
Histology	
SQ	142
Non‐SQ	734
p‐Stage	
IA	429
IB	189
IIA	83
IIB	48
IIIA	101
IIIB	3
IV	23
Smoking history	
−	435
+	441
*EGFR* mutation	
−	524
+	352
*KRAS* mutation	
−	803
+	73
*TP53* mutation	
−	641
+	235
*ALK* mutation	
−	842
+	34
Number of somatic mutations	
0	253
1	451
2	151
3	21

### 3.2 Impact of patients' characteristics on RFS

**Figure 1:**
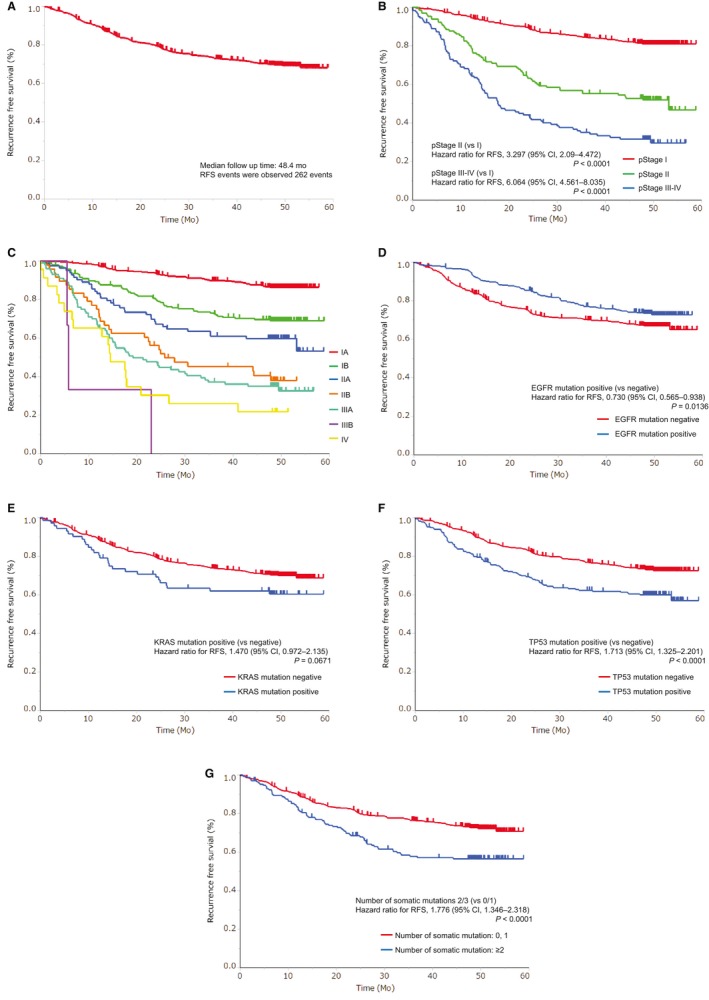
Kaplan–Meier curves of RFS: (A) overall population, (B) according to pathological stage (stage I vs II vs III‐IV), (C) according to pathological stage in detail, (D) according to *EGFR* mutations, (E) according to *KRAS* mutations, (F) according to *TP53* mutations, and (G) according to the number of coexisting somatic mutations

Upon performing univariate analysis, it was found that age (≥70 years), sex (male), histology (squamous carcinoma), pathological (p‐) stage (III‐IV > II > I), smoking history (smoker), *EGFR* mutation (negative), *TP53* mutation (positive), and the number of coexisting mutations (≥2) were factors related to shorter RFS. On the other hand, *KRAS* status and *ALK* rearrangement did not affect RFS (data not shown). Figure 1 shows an RFS curve in the overall population (a) and RFS curves stratified by p‐stage (b and c), *EGFR* (d), *KRAS* (e), and *TP53* genes(f), and the number of coexisting mutations (g). In this observational period, 262 RFS events were recorded. Multivariate analysis showed that the larger number of coexisting mutations (≥2 vs 0 or 1, HR = 2.012, 95% CI: 1.488‐2.695, *P* < .0001), older age (≥70 vs <70 years, HR = 1.583, 95% CI: 1.229‐2.049, *P* = .0004), male gender (male vs female, HR = 1.503, 95% CI: 1.045‐2.170, *P* = .0278), and advanced pathological stage (II vs I, HR = 3.386, 95% CI: 2.447‐4.646, *P* < .0001; III‐IV vs I, HR = 6.307, 95% CI: 4.680‐8.476, *P* < .0001) were significantly associated with shorter RFS (Table 2).

**Table 2:** Prognostic factors for recurrence‐free survival (RFS): multivariate analysis

Factor	HR (95% CI)	*P* value
Age (years)		
≥70	1.583 (1.229‐2.049)	.0004
Sex		
Male	1.503 (1.045‐2.170)	.0278
Histology		
SQ	0.899 (0.627‐1.278)	.5557
p‐Stage		
II (vs I)	3.386 (2.447‐4.646)	<.0001
III/IV (vs I)	6.307 (4.680‐8.476)	<.0001
III/IV (vs II)	1.863 (1.339‐2.605)	.0002
Smoking history		
Smoking habit	0.996 (0.680‐1.464)	.9848
*EGFR* mutation		
Positive	1.017 (0.750‐1.376)	.9108
*KRAS* mutation		
Positive	1.034 (0.665‐1.556)	.8765
*TP53* mutation		
Positive	1.022 (0.769‐1.350)	.8796
Number of somatic mutations		
2/3 (vs 0/1)	2.012 (1.488‐2.695)	<.0001

### 3.3 Impact of patients' characteristics on OS

**Figure 2:**
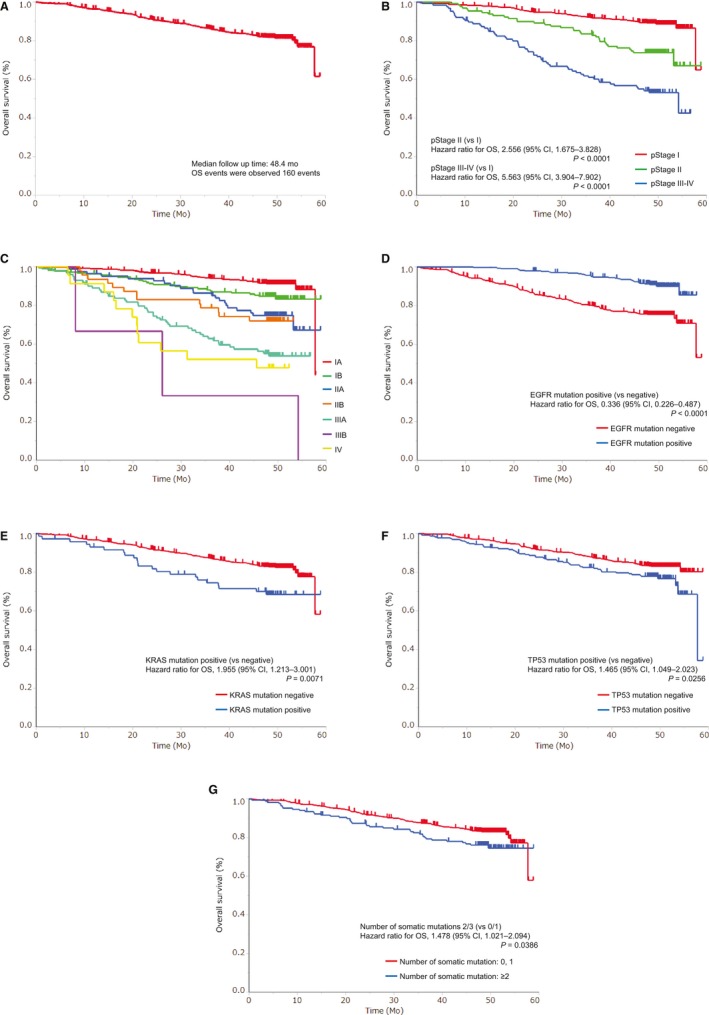
Kaplan‐Meier curves of OS: (A) overall population, (B) according to pathological stage (stage I vs II vs III‐IV), (C) according to pathological stage in detail, (D) according to *EGFR* mutations, (E) according to *KRAS* mutations, (F) according to *TP53* mutations, and (G) according to the number of coexisting somatic mutations

Upon performing univariate analysis, it was found that age (≥70 years), sex (male), histology (squamous carcinoma), and pathological stage (III‐IV > II > I), smoking history (smoker), *EGFR* mutation (negative), *KRAS* mutation (positive), and *TP53* mutation (positive), and the large number of coexisting mutations were factors related to shorter OS. On the other hand, *ALK* rearrangement had no effect on OS (data not shown). Figure 2 shows an OS curve in the overall population (a), and OS curves stratified by p‐stage (b and c), *EGFR* (d), *KRAS* (e), *TP53* (f), and the number of coexisting mutations (g). In this observational period, 160 OS events were recorded. Multivariate analysis showed that *EGFR* mutations (yes vs no, HR = 0.482, 95% CI: 0.309‐0.736, *P* = .0006) were related to longer OS, and the larger number of coexisting mutations (≥2 vs 0 or 1, HR = 1.695, 95% CI: 1.143‐2.467, *P* = .0093), older age (≥70 vs <70 years, HR = 1.932, 95% CI: 1.385‐2.726, *P* < .0001), and advanced pathological stage (II vs I, HR = 2.209, 95% CI: 1.431‐3.347, *P* < .0001; III‐IV vs I, HR = 5.286, 95% CI: 3.682‐7.566, *P* < .0001) were related to shorter OS (Table 3).

**Table 3:** Prognostic factors for overall survival (OS): multivariate analysis

Factor	HR (95% CI)	*P* value
Age (years)		
≥70	1.932 (1.385‐2.726)	<.0001
Sex		
Male	1.157 (0.730‐1.864)	.5403
Histology		
SQ	1.330 (0.883‐1.990)	.1706
p‐Stage		
II (vs I)	2.209 (1.431‐3.347)	.0005
III/IV (vs I)	5.286 (3.682‐7.566)	<.0001
III/IV (vs II)	2.393 (1.567‐3.713)	<.0001
Smoking history		
Smoking habit	1.437 (0.865‐2.380)	.1609
*EGFR* mutation		
Positive	0.482 (0.309‐0.736)	.0006
*KRAS* mutation		
Positive	1.253 (0.753‐2.002)	.3725
*TP53* mutation		
Positive	0.820 (0.570‐1.170)	.2764
Number of somatic mutations		
2/3 (vs 0/1)	1.695 (1.143‐2.467)	.0093

## 4 DISCUSSION

In this prospective analysis, a smaller number of coexisting mutations were associated with longer RFS and OS, implying that multiple mutations are indicative of cancer aggressiveness, thereby resulting in a high relapse rate. *EGFR* mutation positivity was associated with longer OS, suggesting that the prognosis of *EGFR* mutation‐related lung cancer could be improved by *EGFR*‐targeted therapy, which may also apply to incidences of resected NSCLC. To the best of our knowledge, the present research is one of the first report prospectively showing that the number of coexisting mutations affects RFS and OS, and the *EGFR* mutation status has a significant impact on OS in resected NSCLC.

In previous reports, *KRAS* mutation was not a significant prognostic factors in resected early‐stage NSCLC,^21^ with similar results recently reported for *TP53*.^16^ Although *EGFR* mutation is associated with longer survival in advanced diseases, some previous study reported that such results have been inconsistent in surgical series.^22^ Furthermore, in a prior report, *EGFR*, *KRAS,* and *EGFR*/*KRAS* plus *TP53* co‐mutations were not significant prognostic markers in early‐stage resected NSCLC.^23^ The study retrospectively analyzed the impact of *EGFR*, *KRAS*, and *TP53* mutations on OS using four key trials of early‐stage resected NSCLC. The reason why these factors did not affect OS might be that there was no chemotherapy effectively targeting *KRAS* and *TP53* mutations, and *EGFR*‐TKIs were not yet in clinical use at the time. Here, we have shown that *EGFR* mutation positivity was associated with longer OS, likely reflecting that *EGFR*‐TKIs were used as a standard therapy in patients harboring *EGFR* mutations during the period of the present study.^5^, ^6^, ^7^ Although erlotinib, used as an adjuvant agent in adjuvant therapy, did not improve OS even in *EGFR* mutation‐positive subgroup of 161 (16.5%) patients in the prior trial (RADIANT)^24^; it was interpreted that *EGFR*‐TKI therapy after recurrence effectively prolonged survival.

On the other hand, a smaller number of coexisting mutations were associated with longer RFS and OS in the present study. Recently, it has been found that, in the evolution of tumors, early founder (clonal or trunk) somatic mutational events that drive tumorigenesis develop as clonal mutations, genome doubling events often occur early in tumor evolution in the trunk of the evolutionary tree, and subclonal driver events may follow after genome doubling in the branches of the evolutionary tree of the tumor.^25^ Zhang et al applied multiregion, whole‐exome sequencing to specimens from eleven patients with early stage lung adenocarcinoma, and they demonstrated associations of the numbers of subclones in the tumor with relapse.^26^ They showed that larger subclonal mutation fractions may be correlated with an increased likelihood of postsurgical relapse in localized lung adenocarcinoma patients. Although larger prospective trials are needed to confirm the results, the present trial, a large‐scale prospective study that analyzed somatic mutations in early stage NSCLC, provides robust support for these observations.

In this study, pathological earlier stage and younger age were correlated with longer RFS and OS, and the stage of cancer was the most crucial factor affecting RFS and OS. These results demonstrate that progression of cancer, including metastasis, is more influential than the status of coexistent mutations. Figures 1 and 2(b and c) show that the pathological stage is associated not only with OS but also, clearly, with RFS. TNM classification version 7^18^ is based on a retrospective examination that accumulated a large quantity of data,^27^ and it was reported by Chansky et al (2009) that the analysis of the classification confirmed age and sex as important prognostic factors, while histology was less important in surgically resected NSCLC. Finally, they showed pathologic TNM category as the most significant prognostic factor. This study is the first to have proven the accuracy of TNM classification version 7 in the prospective manner and to have demonstrated that the pathological stage is the independent prognostic factor irrespective of patient background and somatic mutations. In addition, age was also confirmed as an important prognostic factor. Although older age was related to higher recurrence rate, the relationship may be the same as the correlation between age and morbidity.

Limitations of the present research include a small number of recurrence events and death events, and the relatively short observation period (4 years). There were a considerable number of stage I patients whose prognosis has become better, that is, the relapse rate is quite low due to the progress of diagnostic techniques and developments in improved surgical techniques, resulting in decreased incidences of relapse and death. Although the present study is the largest prospective trial to analyze correlations between prognosis and somatic mutations, a considerable part of the results lacks enough statistical power, and they should be interpreted with caution and need further validation in a prospective study. However, the prognostic information was collected exactly as planned with no missing data, and we have successfully demonstrated the correlations between coexisting mutations and prognosis on multivariate analysis. Another limitation is that the entire genome sequences were not examined, and not all somatic mutations were tested. However, major somatic mutations in genes related to cancer incidence were covered in our analyses, and clinically meaningful somatic mutations were sufficiently tested and their impacts and those of coexisting mutations on prognosis were also effectively examined.

In conclusion, this prospective, observational study showed that a smaller number of coexisting mutations, earlier stage, sex (female), and younger age were associated with longer RFS, while *EGFR* mutation positivity was significantly associated with improved OS, as well as earlier stage, a smaller number of coexisting mutations, and younger age, in resected NSCLC. The outcomes of the JME study provide valuable information on the impact of somatic mutations and coexisting mutations on RFS and OS, and further prospective studies are warranted to better understand the impacts of somatic mutations and coexisting mutations.
